# Supplementary material for: Sunken Riches: Ascomycete Diversity in the Western Mediterranean Coast through Direct Plating and Flocculation, and Description of Four New Taxa
Source: J Fungi (Basel). 2024 Apr 11;10(4):281. doi: 10.3390/jof10040281 (PMC11051201; doi:10.3390/jof10040281)
Supplement: Supplementary file 1 [file jof-10-00281-s001.zip › jof-2896513-supplementary.pdf]

Table S1: Collection details of the strains detected in marine sediments and GenBank accession numbers of the barcodes used for identification.

Molecular ID	Family	**FMR**	**Beach**	Depth	Agar media	ITS	LSU	TUB	TEF	RPB2	SSU
Acremonium egyptiacum	*Bionectriaceae*	**19979**	**Arrabassada**	13	DRBC	PP273903			PP392587		
Acrophialophora jodhpurensis	*Chaetomiaceae*	**19999**	**Arrabassada**	20	SWMEA3%	PP273913					
		**20081**	**Arrabassada**	20	SWMEA3%	PP273920				PP412563	
Amphichorda littoralis	*Bionectriaceae*	**19404**	**Miracle**	20	SWMEA3%	OQ942924	OQ943161				
		**19611**	**Miracle**	20	DRBC	OQ942926	OQ943163				
		**20066**	**Arrabassada**	27	SWMEA3%	PP344593	PP342585				
		**20067**	**Arrabassada**	20	SWMEA3%	OQ942927	OQ943164				
		**20149**	**Arrabassada**	20	SWMEA3%	OQ942928	OQ943165				
		**20291**	**Arrabassada**	20	SWMEA3%	PP344594	PP342586				
		**20368**	**Arrabassada**	20	SWMEA3%	PP344595	PP342587				
Aphanoascus crassitunicatus	*Onygenaceae*	**20176**	**Arrabassada**	13	PDA+C	PP273949					
Aphanoascus fulvescens	*Onygenaceae*	**19946**	**Arrabassada**	27	SWMEA3%	PP273899					
		**20116**	**Arrabassada**	27	SWMEA3%	PP273928					
Arachnomyces sp.	*Arachnomycetaceae*	**20163**	**Arrabassada**	27	SWMEA3%	PP273940					
Arthrographis curvata	*Eremomycetaceae*	**19557**	**Miracle**	20	DRBC	PP273869					
		**20084**	**Arrabassada**	20	SWMEA3%	PP273921					
Arthrographis kalrae	*Eremomycetaceae*	**20074**	**Arrabassada**	20	PDA+C	PP273917					
Aspergillus calidoustus	*Aspergillaceae*	**20172**	**Arrabassada**	20	DRBC			PP431576			
Aspergillus chevalieri	*Aspergillaceae*	**19829**	**Miracle**	27	SWMEA3%			PP431577			
		**19831**	**Miracle**	13	DRBC			PP431578			
		**20122**	**Miracle**	27	DRBC			PP431579			
		**20123**	**Miracle**	20	DRBC			PP431580			
		**19837**	**Miracle**	20	SWMEA3%			PP431581			
		**19838**	**Miracle**	20	SWMEA3%			PP431582			
Aspergillus intermedius	*Aspergillaceae*	**20167**	**Miracle**	20	SWMEA3%			PP431583			
Aspergillus montevidensis	*Aspergillaceae*	**20076**	**Arrabassada**	20	SWMEA3%	PP273919		PP431584			
Aspergillus pseudoglaucus	*Aspergillaceae*	**19830**	**Miracle**	27	SWMEA3%			PP431585			
		**20121**	**Miracle**	27	SWMEA3%			PP431586			
		**19832**	**Miracle**	13	SWMEA3%			PP431587			
Aspergillus quadrilineatus	*Aspergillaceae*	**19833**	**Miracle**	27	DRBC			PP431588			
		**19467**	**Miracle**	20	DRBC			PP431589			
Aspergillus terreus	*Aspergillaceae*	**19949**	**Arrabassada**	20	SWMEA3%			PP431590			

		**20001**	**Arrabassada**	20	DRBC			PP431591			
Botryotrichum spirotrichum	*Chaetomiaceae*	**19980**	**Arrabassada**	27	SWMEA3%					PP412564	
Byssoonygena ceratinophila	*Onygenaceae*	**19558**	**Miracle**	13	DRBC	PP273870	PP342588				
Chaetomium sp.-1	*Chaetomiaceae*	**19623**	**Miracle**	27	DRBC	PP340477					
		**19939**	**Arrabassada**	13	DRBC	PP273895					
Chantransiopsis cf. decumbens	*Incertae sedis*	**19986**	**Arrabassada**	27	SWMEA3%	PP273906	PP342589				PP340176
Cladophialophora inmunda	*Herpotrichiellaceae*	**20179**	**Miracle**	20	DRBC	PP273952					
Cladophialophora saturnica	*Herpotrichiellaceae*	**19605**	**Miracle**	13	PDA+C	PP273876					
Cladosporium cladosporioides	*Cladosporiaceae*	**20182**	**Miracle**	20	DRBC				PP386376		
		**20181**	**Miracle**	20	SWMEA3%				PP386377		
Cladosporium sp.	*Cladosporiaceae*	**20180**	**Miracle**	6	SWMEA3%				PP386383		
Collariella pachypodioides	*Chaetomiaceae*	**19944**	**Arrabassada**	27	DRBC	PP273897				PP412565	
Cucurbidothis pithyophila	*Cucurbitariaceae*	**19846**	**Miracle**	20	DRBC					PP412566	
Emericellopsis maritima	*Bionectriaceae*	**19565**	**Miracle**	6	SWMEA3%	PP273874					
		**20087**	**Miracle**	27	PDA+C	PP273923	PP342590		PP392588	PP412567	
		**20164**	**Arrabassada**	27	SWMEA3%	PP273941			PP392589	PP412568	
Emericellopsis microspora	*Bionectriaceae*	**19396**	**Miracle**	27	SWMEA3%	PP273851					
Emericellopsis minima	*Bionectriaceae*	**19461**	**Miracle**	20	DRBC	PP273853	PP342591		PP392590		
Emericellopsis salmosynnemata	*Bionectriaceae*	**19609**	**Miracle**	6	DRBC	PP273880				PP412581	
		**20160**	**Arrabassada**	27	SWMEA3%	PP273938				PP412569	
Exophiala littoralis	*Herpotrichiellaceae*	**19606**	**Miracle**	6	DRBC	PP273877	PP342592	PP431592			
		**19607**	**Miracle**	6	DRBC	PP273878	PP342593	PP431593			
Exophiala xenobiotica	*Herpotrichiellaceae*	**19604**	**Miracle**	27	SWMEA3%	PP273875					
Fusarium falciforme	*Nectriaceae*	**20152**	**Arrabassada**	13	SWMEA3%				PP386378		
Fusarium solani	*Nectriaceae*	**19854**	**Arrabassada**	27	SWMEA3%				PP386379		
Gamsia columbina	*Microascaceae*	**19393**	**Miracle**	27	DRBC	PP273849					
		**19556**	**Miracle**	13	SWMEA3%	PP273868					
		**19853**	**Arrabassada**	20	PDA+C	PP273894					
		**19991**	**Arrabassada**	20	PDA+C	PP273910					
		**20175**	**Arrabassada**	13	PDA+C	PP273948					
Gliomastix masseei	*Bionectriaceae*	**19948**	**Arrabassada**	20	SWMEA3%	PP273901					
Gymnascella dankaliensis	*Gymnoascaceae*	**19625**	**Miracle**	27	PDA+C	PP273888					
		**19616**	**Miracle**	20	SWMEA3%	PP273883	PP342594				
		**20162**	**Arrabassada**	27	PDA+C	PP273939					
Gymnoascoideus sp.	*Gymnoascaceae*	**19992**	**Arrabassada**	27	PDA+C	PP273911					

Gymnoascus longitrichus	*Gymnoascaceae*	**19624**	**Miracle**	20	PDA+C	PP273887					
Gymnoascus reessii	*Gymnoascaceae*	**19395**	**Miracle**	27	SWMEA3%	PP273850					
Lophotrichus fimeti	*Microascaceae*	**20072**	**Arrabassada**	20	SWMEA3%	PP273915	PP342595				
Malbranchea ostraviensis	*Malbrancheaceae*	**20173**	**Arrabassada**	27	DRBC	PP273946					
Malbranchea zuffiana	*Malbrancheaceae*	**20086**	**Miracle**	20	PDA+C	PP273922	PP342596				
Malbranchea sp.-1	*Malbrancheaceae*	**19564**	**Miracle**	27	DRBC	PP344596					
		**20150**	**Arrabassada**	27	PDA+C	PP344597					
Malbranchea sp.-2	*Malbrancheaceae*	**19403**	**Miracle**	27	SWMEA3%	PP344598					
Malbranchea sp.-3	*Malbrancheaceae*	**20151**	**Arrabassada**	27	PDA+C	PP344599					
Microascus trigonosporus	*Microascaceae*	**19945**	**Arrabassada**	27	SWMEA3%	PP273898		PP431597			
Narasimhella hialinospora	*Gymnoascaceae*	**20283**	**Arrabassada**	20	DRBC	PP273953					
Narasimhella poonensis	*Gymnoascaceae*	**19620**	**Miracle**	27	SWMEA3%	PP273885	PP342597				
		**19613**	**Miracle**	27	SWMEA3%	PP273882	PP342598				
		**19621**	**Miracle**	27	SWMEA3%	PP273886	PP342599				
		**19978**	**Arrabassada**	27	PDA+C	PP273902					
		**20145**	**Arrabassada**	27	PDA+C	PP273931					
Nigrocephalum paracollariferum	*Plectosphaerellaceae*	**19852**	**Arrabassada**	27	SWMEA3%	PP273893					
		**20069**	**Arrabassada**	20	SWMEA3%	PP273914	PP342600		PP392591	PP412570	
		**20174**	**Arrabassada**	13	SWMEA3%	PP273947	PP342601		PP392592	PP412571	
Onygenal no ID-1	? (*Onygenales*)	**19619**	**Miracle**	27	DRBC						PP459576
Onygenal no ID-2	? *(Onygenales)*	**19614**	**Miracle**	13	SWMEA3%						PP459575
Paraphaeospaheria sp.	*Didymosphaeriaceae*	**20158**	**Arrabassada**	6	DRBC	PP273937	PP342602				
Parapyrenis maritima	*Requienellaceae*	**19985**	**Arrabassada**	27	SWMEA3%	PP273905					
Parasarocladium wereldwijsianum	*Sarocladiaceae*	**20177**	**Miracle**	27	SWMEA3%	PP273950	PP342603				
		**19608**	**Miracle**	27	SWMEA3%	PP273879					
		**20147**	**Arrabassada**	27	SWMEA3%	PP273933					
		**20146**	**Arrabassada**	27	SWMEA3%	PP273932					
Parathielavia kuwaitensis	*Chaetomiaceae*	**19555**	**Miracle**	27	SWMEA3%	PP273867				PP412572	
Penicillium antarcticum	*Aspergillaceae*	**20433**	**Arrabassada**	27	SWMEA3%			PP431598			
		**20434**	**Arrabassada**	20	DRBC			PP431599			
		**20171**	**Arrabassada**	27	PDA+C			PP431600			
Penicillium brevicompactum	*Aspergillaceae*	**19401**	**Miracle**	27	SWMEA3%			PP431601			
Penicillium canescens	*Aspergillaceae*	**20002**	**Arrabassada**	27	SWMEA3%			PP431602			
Penicillium chrysogenum	*Aspergillaceae*	**20125**	**Miracle**	13	DRBC			PP431603			
Penicillium citreosulfuratum	*Aspergillaceae*	**19943**	**Arrabassada**	27	SWMEA3%			PP431604			

Penicillium egyptiacum	*Aspergillaceae*	**20119**	**Miracle**	27	SWMEA3%			PP431605			
		**20120**	**Miracle**	27	SWMEA3%			PP431606			
		**19463**	**Miracle**	27	DRBC			PP431607			
		**19835**	**Miracle**	27	SWMEA3%			PP431608			
		**19836**	**Miracle**	20	DRBC			PP431609			
		**20124**	**Miracle**	6	DRBC			PP431610		PP412573	
Penicillium nalgiovense	*Aspergillaceae*	**19987**	**Arrabassada**	20	SWMEA3%			PP431611			
		**20090**	**Arrabassada**	20	SWMEA3%			PP431612			
Penicillium rubens	*Aspergillaceae*	**19402**	**Miracle**	27	SWMEA3%			PP431613			
		**19405**	**Miracle**	20	SWMEA3%			PP431614			
		**19406**	**Miracle**	6	SWMEA3%	PP273852		PP431615			
Preussia procaviae	*Sporormiaceae*	**19839**	**Miracle**	27	SWMEA3%	PP273890					
Preussia similis	*Sporormiaceae*	**19464**	**Miracle**	27	SWMEA3%	PP273854	PP342604				
Preussia sp.-1	*Sporormiaceae*	**19850**	**Miracle**	20	DRBC	PP344600	PP342618				
Preussia sp.-2	*Sporormiaceae*	**19618**	**Miracle**	27	DRBC	PP344601	PP342619				
		**20127**	**Miracle**	27	DRBC	PP344602	PP342620				
		**20161**	**Arrabassada**	20	SWMEA3%	PP344603	PP342621				
Preussia sp.-3	*Sporormiaceae*	**19842**	**Miracle**	20	SWMEA3%	PP344604	PP342622				
Pseudeurotium desertorum	*Pseudeurotiaceae*	**19617**	**Miracle**	20	SWMEA3%	PP273884	PP342605				
Pseudeurotium ovale	*Pseudeurotiaceae*	**19554**	**Miracle**	20	DRBC	PP273866					
		**19553**	**Miracle**	20	DRBC	PP273865					
		**19984**	**Miracle**	20	DRBC	PP273904					
		**20165**	**Arrabassada**	27	DRBC	PP273942					
		**20075**	**Arrabassada**	20	SWMEA3%	PP273918					
		**20155**	**Arrabassada**	27	SWMEA3%	PP273936					
		**20153**	**Arrabassada**	27	SWMEA3%	PP273934					
		**20285**	**Arrabassada**	27	SWMEA3%	PP273954					
		**20117**	**Arrabassada**	20	DRBC	PP273929					
Pseudogymnoascus pannorum	*Pseudeurotiaceae*	**19465**	**Miracle**	27	PDA+C	PP273855					
Pseudohumicola sp.	*Chaetomiaceae*	**20111**	**Arrabassada**	13	SWMEA3%	PP273925					
Queenslandipenidiella verrucosa	*Teratosphaeriaceae*	**19473**	**Miracle**	20	DRBC	PP273856	PP342606				
		**19474**	**Miracle**	20	PDA+C	PP273857	PP342607				
		**19475**	**Miracle**	20	PDA+C	PP273858	PP342608				
		**19476**	**Miracle**	20	PDA+C	PP273859	PP342609				
		**19477**	**Miracle**	13	DRBC	PP273860	PP342610	PP431594	PP386380		

		**19478**	**Miracle**	13	SWMEA3%	PP273861	PP342611		PP386381		
		**19479**	**Miracle**	13	PDA+C	PP273862	PP342612		PP386382		
		**19480**	**Miracle**	13	PDA+C	PP273863					
		**19481**	**Miracle**	6	PDA+C	PP273864					
Roussoella padinae	*Thyridariaceae*	**19988**	**Arrabassada**	27	SWMEA3%	PP273907				PP412574	
		**19989**	**Arrabassada**	6	SWMEA3%	PP273908				PP412575	
		**20148**	**Arrabassada**	13	DRBC					PP412576	
Scedosporium apiospermum	*Microascaceae*	**19940**	**Arrabassada**	20	PDA+C	PP273896		PP431616			
Scedosporium boydii	*Microascaceae*	**19562**	**Miracle**	27	DRBC	PP273873		PP431617			
		**20369**	**Arrabassada**	27	DRBC	PP273958					
		**20112**	**Arrabassada**	27	DRBC	PP273926		PP431618			
		**20169**	**Arrabassada**	27	SWMEA3%	PP273944		PP431619			
Scedosporium dehoogii	*Microascaceae*	**19561**	**Miracle**	27	SWMEA3%	PP273872		PP431620			
		**19612**	**Miracle**	20	SWMEA3%	PP273881		PP431621			
Schizochlamydosporiella marina	*Schizotheciaceae*	**20114**	**Arrabassada**	27	DRBC	PP273927	PP342613	PP431595		PP412577	
Schizothecium carpinicola	*Schizotheciaceae*	**20154**	**Arrabassada**	27	DRBC	PP273935					
Scytalidium lignicola	*Incertae sedis*	**19849**	**Miracle**	27	SWMEA3%	PP273892					
		**20170**	**Arrabassada**	27	SWMEA3%	PP273945					
Sporothrix sp.	*Ophiostomataceae*	**20288**	**Arrabassada**	27	PDA+C	PP273955					
Stachybotrys chlorohalonatus	*Stachybotryaceae*	**19626**	**Miracle**	20	SWMEA3%	PP273889					
		**19982**	**Arrabassada**	27	SWMEA3%	PP340478					
		**19947**	**Arrabassada**	27	PDA+C	PP273900					
		**20370**	**Arrabassada**	27	SWMEA3%	PP273959					
Stolonocarpus sp.	*Chaetomiaceae*	**19559**	**Miracle**	27	SWMEA3%	PP273871	PP342614				
		**20178**	**Miracle**	27	SWMEA3%	PP273951	PP342615				
Subuliphorum camptosporum	*Clavicipitaceae*	**20073**	**Miracle**	13	PDA+C	PP273916	PP342616				
Talaromyces assiutensis	*Trichocomaceae*	**20085**	**Arrabassada**	13	DRBC	PP341503					
		**20156**	**Arrabassada**	20	DRBC			PP431622			
Talaromyces liani	*Trichocomaceae*	**20157**	**Miracle**	27	DRBC			PP431623			
		**20089**	**Miracle**	13	DRBC			PP431624			
		**20432**	**Arrabassada**	27	DRBC			PP431625			
		**20290**	**Arrabassada**	13	DRBC			PP431626			
Talaromyces trachispermum	*Trichocomaceae*	**19981**	**Arrabassada**	20	DRBC			PP431627			
Talaromyces ucrainicus	*Trichocomaceae*	**19394**	**Miracle**	27	DRBC			PP431628			
		**19855**	**Arrabassada**	13	DRBC			PP431629			

Talaromyces wortmannii	*Trichocomaceae*	**19397**	**Miracle**	20	DRBC			PP431630			
		**19398**	**Miracle**	20	DRBC			PP431631			
		**19399**	**Miracle**	13	DRBC			PP431632			
		**19400**	**Miracle**	27	DRBC			PP431633			
Talaromyces sp.-1	*Trichocomaceae*	**19610**	**Miracle**	20	DRBC			PP431634		PP412578	
		**20079**	**Arrabassada**	13	DRBC			PP431635			
Triadelphia moubasheri	*Triadelphiaceae*	**19840**	**Miracle**	6	SWMEA3%	PP273891					
Verruciconidia verruculosa	*Bionectriaceae*	**20166**	**Arrabassada**	27	SWMEA3%	PP273943				PP412579	
Waltergamsia fusidioides	*Bionectriaceae*	**20110**	**Arrabassada**	6	SWMEA3%	PP273924				PP412580	
		**20289**	**Arrabassada**	6	SWMEA3%	PP273956					
		**20292**	**Arrabassada**	6	SWMEA3%	PP273957					
Westerdykella dispersa	*Sporormiaceae*	**20118**	**Arrabassada**	27	SWMEA3%	PP273930	PP342617	PP431596			
Zopfiella sp.-1	*Lasiosphaeriaceae*	**19995**	**Arrabassada**	27	SWMEA3%	PP273912					
		**19990**	**Arrabassada**	27	SWMEA3%	PP273909					

Table S2: Accession numbers of the strains included in the phylogenetic analyses.

Species	Strain number	GenBank accession numbers				References
		ITS	LSU	*tef*1-a	*rpb*2	
Exophiala lamphunensis	CMU 404	NR_184985	NA	NA	NA	[101]
Exophiala xenobiotica	CBS 118157	NR_111203		NA	NA	[102]
Exophiala saxicola	CMU 415	NR_184987	NA	NA	NA	[101]
Exophiala lapidea	CMU 409	NR_184986	NA	NA	NA	[101]
Exophiala campbellii	NCPF2274_T	LT594703	LT594760	NA	NA	[103]
	NCPG7936	MN091928	MN091927	NA	NA	[104]
Exophiala arunalokei	NCCPF106033	MW724320	NA	NA	NA	[105]
Exophiala italica	MFLUCC 16-0245	KY496744	KY496723	NA	NA	[106]
Exophiala hongkongensis	HKU 32	NR_111671	NG_059264	NA	NA	[102,65]
Exophiala polymorpha	CBS 138920	KP070764	NG_059237	NA	NA	[107]
Exophiala nishimurae	CBS 101538	NR_137092	KX712351	NA	NA	[65,108]
Exophiala spinifera	CBS 899.68	NR_111131	Westertdijk	NA	NA	[102]
Exophiala exophialae	CBS 668.76_T	NR_111130	NG_059252	NA	NA	[102,108]
Exophiala spartinae	CBS 142672_T	NR_174648	NA	NA	NA	[109]
Exophiala jeanselmei	CBS 507.90_T	AY156963	MH873915	NA	NA	[61,110]
Exophiala oligosperma	CBS 725.88_T	NR_111134	NG_059201	NA	NA	[102,111]
	CBS 265.49_T	MH856519	MH868049	NA	NA	[110,112]
Exophiala pseudooligosperma	YMF 1.6741_T	MW616557	MW616559	NA	NA	[110]
Rhinocladiella basitona	CBS 101460_T	NR_111135	NG_057783	NA	NA	[102,113]
Rhinocladiella similis	PW3041	LC158611	LC158635	NA	NA	[13]
Exophiala bergeri	CBS 353.52_T	MH857080	NG_059199	NA	NA	[110,111]
Exophiala ellipsoidea	CGMCC 3.17348_T	NR_172238	KP347956	NA	NA	[115]
Exophiala sideris	CBS 121818_T	NR_111553	NA	NA	NA	[102]
Exophiala capensis	CBS 128771_T	NR_121493	MH876538	NA	NA	[102,110]
Exohiala nigra	CBS 535.94_T	NR_154974	NG_059253	NA	NA	[108,116]
Exophiala dehoogii	CBS 149779_T	ON009858	ON009938	NA	NA	[45]
Exophiala palmae	UPCB 86822_T	NR_158414	NG_064428	NA	NA	[117]
Nigrocephalum collariferum	CBS 124586_T	MH863392	MH874911	LR026486	LR026193	[46,110]
	CBS 124585	FJ765365	LR025928	LR026485	LR026192	[46,110]
Wallrothiella gmelinae	CBS 142520_T	KY979753	KY979808	NA	NA	[118]
Wallrothiela subiculosa	JCM 23118	AB540576	AB540502	NA	NA	[119]
Chlamydosporiella restricta	CBS 178.40_T	MH856081	MH867572	LR026395	LR026122	[46,110]
	CBS 119.97	LR026691	LR025820	LR026393	LR026120	[46]
Stachylidium bicolor	CBS 121802_T	LR026834	LR025972	LR026532	NA	[46]
Stachylidium pallidum	DAOMC 226658	LR026838	GU180651	LR026534	LR026228	[46,120]
	BCC 79031	LR026835	LR025973	LR026533	LR026227	[46]
Paramusicillium asperulatum	CBS 120158_T	LR026792	LR025930	LR026487	LR026194	[46]
Musicillium theobromae	CBS 968.72_T	LR026773	LR025907	LR026468	LR026178	[46]
	CBS 397.58	LR026772	LR025906	LR026467	LR026177	[46]
Musicillium elettariae	CBS 252.80_T	LR026765	LR025899	LR026462	LR026172	[46]
	CBS 110322	LR026766	LR025900	LR026463	NA	[46]
Musicillium tropicale	CBS 120009_T	LR026783	LR025917	LR026477	LR026186	[46]
	CBS 395.58	LR026779	LR025913	LR026474	LR026182	[46]
Phialoparvum bifurcatum	CBS 299.70B_T	LR026793	LR025931	LR026488	LR026195	[46]
Plectosphaerella cucumerina	CBS 137.33_T	LR026797	LR025935	LR026492	LR026198	[46]
Queenslandipenidiella kurandae	CBS 121715_T	KF901538	KF901860	NA	NA	[44]
Devriesia thermodurans	CBS 115878_T	KF442506	NG_059078	NA	NA	[121,122]
Devriesia tardicrescens	CBS 128770_T	NR_137771	NG_059091	NA	NA	[123]

Baudoinia compniacensis	CBS 123032	MH863266	MH874790	NA	NA	[110]
Baudoinia antilliensis	UAMH 10810_T	NR_153616	NG_058686	NA	NA	[124]
Baudoinia orientalis	UAMH 10814_T	NR_153613	KT186490	NA	NA	[124]
Apenidiella strumelloidea	CBS 114484_T	MH862966	KF937229	NA	NA	[44,110]
Apenidiella foetida	FMR 17266	NR_165516	NG_067803	NA	NA	[125]
Oleoguttula mirabilis	CCFEE 5523_T	NR_144974	KF310031	NA	NA	[44]
Parapenidiella tasmaniensis	CBS 111687_T	DQ267591	KF901843	NA	NA	[44,126]
Parapenidiella pseudotasmaniensis	CBS 124991_T	MH863440	MH874943	NA	NA	[110]
Penidiella columbina	CBS 486.80_T	MH861288	MH873053	NA	NA	[110]
Teratosphaeria fibrillosa	CBS 121707_T	MH863138	MH874689	NA	NA	[110]
Teratosphaeria macowanii	CBS 122901_T	MH863257	MH874781	NA	NA	[110]
Readeriella tasmanica	CBS 125002_T	MH863448	KF902116	NA	NA	[44,110]
Readeriella mirabilis	CBS 125000_T	KF901549	KF901871	NA	NA	[44]
Xenopenidiella rigidophora	CBS 314.95_T	NR_175015	MH874156	NA	NA	[110,121]
Suberoteratosphaeria pseudosuberosa	CBS 118911_T	KF901786	KF902144	NA	NA	[44]
Suberoteratosphaeria suberosa	CBS 436.92_T	KF901623	KF901949	NA	NA	[44]
Phaeothecoidea eucalypti	CBS 120831_T	KF901526	KF901848	NA	NA	[44]
Pseudoteratosphaeria flexuosa	CBS 111012_T	KF901755	KF902110	NA	NA	[44]
Pseudoteratosphaeria perpendicularis	CBS 118367_T	KF901637	KF901972	NA	NA	[44]
Myrtapenidiella eucalypti	CBS 123246_T	KF901772	KF902130	NA	NA	[44]
Myrtapenidiella tenuiramis	CBS 124993_T	MH863441	GQ852626	NA	NA	[110,127]
Ramularia endophylla	CBS 113265_T	KF251220	KF251723	NA	NA	[44]
Ramularia eucalypti	CBS 120726_T	KF901666	KF902006	NA	NA	[44]
Zopfiella tardifaciens	CBS 670.82_T	MK926855	MK926855	NA	MK876817	[128]
Cercophora newfieldiana	SMH 3303	NA	AY780062	NA	AY780167	[129]
Cercophora thailandica	MFLUCC 12-0845_T	KU940139	KU863127	NA	KU940176	[130]
Apodus deciduus	CBS 506.70_T	AY681199	AY681165	NA	NA	[131]
Podospora intestinacea	CBS 113106	AY999121	AY999104	NA	NA	[132]
Arnium cirriferum	CBS 120041	NA	KF557673	NA	NA	[133]
Zopfiella erostrata	CBS 255.71	AY999133	AY999110	NA	NA	[132]
Apiosordaria microcarpa	CBS 692.82_T	MK926841	MK926841	NA	MK876803	[128]
Immersiella caudata	CBS 606.72	AY999135	AY999113	NA	DQ368646	[129,134]
Amesia atrobrunnea	CBS 379.66_T	MH858833	MH870470	NA	KX976798	[110,135]
Triangularia bambusae	CBS 352.33_T	MK926868	MK926868	NA	MK876830	[128]
Zygopleurage zygospora	SMH 4219	NA	AY346306	NA	NA	[136]
Podospora bullata	CBS 115576_T	DQ166960	MH874548	NA	NA	[110,137]
Pseudoechria longicolis	CBS 368.52_T	MK926847	MK926847	NA	MK876809	[128]
Pseudoechria prolifica	CBS 250.71_T	MK926848	MK926848	NA	MK876810	[128]
Pseudoechria decidua	CBS 254.71_T	MK926842	MK926842	NA	MK876804	[128]
Rinaldiella pentagonospora	CBS 132344_T	MH866007	KP981442	NA	KP981625	[47,110]
Morinagamyces vermicularis	CBS 303.81_T	MT904879	KP981427	NA	KP981609	[138]
Immersiella caudata	SMH 3298	NA	AY436407	NA	AY780161	[129,134]
Immersiella immersa	SMH 4104	NA	AY436409	NA	AY780181	[129,134]
Jugulospora rotula	CBS 110112	NA	KP981434	NA	KP981617	[47]
Jugulospora carbonaria	ATCC 34567	NA	AY346302	NA	AY780196	[129,136]
Cercophora mirabilis	CBS 120402	MT784128	KP981429	NA	KP981611	[47]
Lundqvistomyces tanzaniensis	TRTC 51981_T	MH862260	AY780081	NA	AY780197	[110,129]
Lundqvistomyces karachiensis	CBS 657.74_T	MK926850	KP981447	NA	KP981630	[47,128]
Schizothecium curvisporum	ATCC 36709	NA	AY346300	NA	AY780192	[132]
Schizothecium selenosporum	CBS 109403_T	MK926849	MK926849	NA	MK876811	[128]
Schizothecium inaequale	CBS 356.49_T	MK926846	MK926846	NA	MK876808	[128]

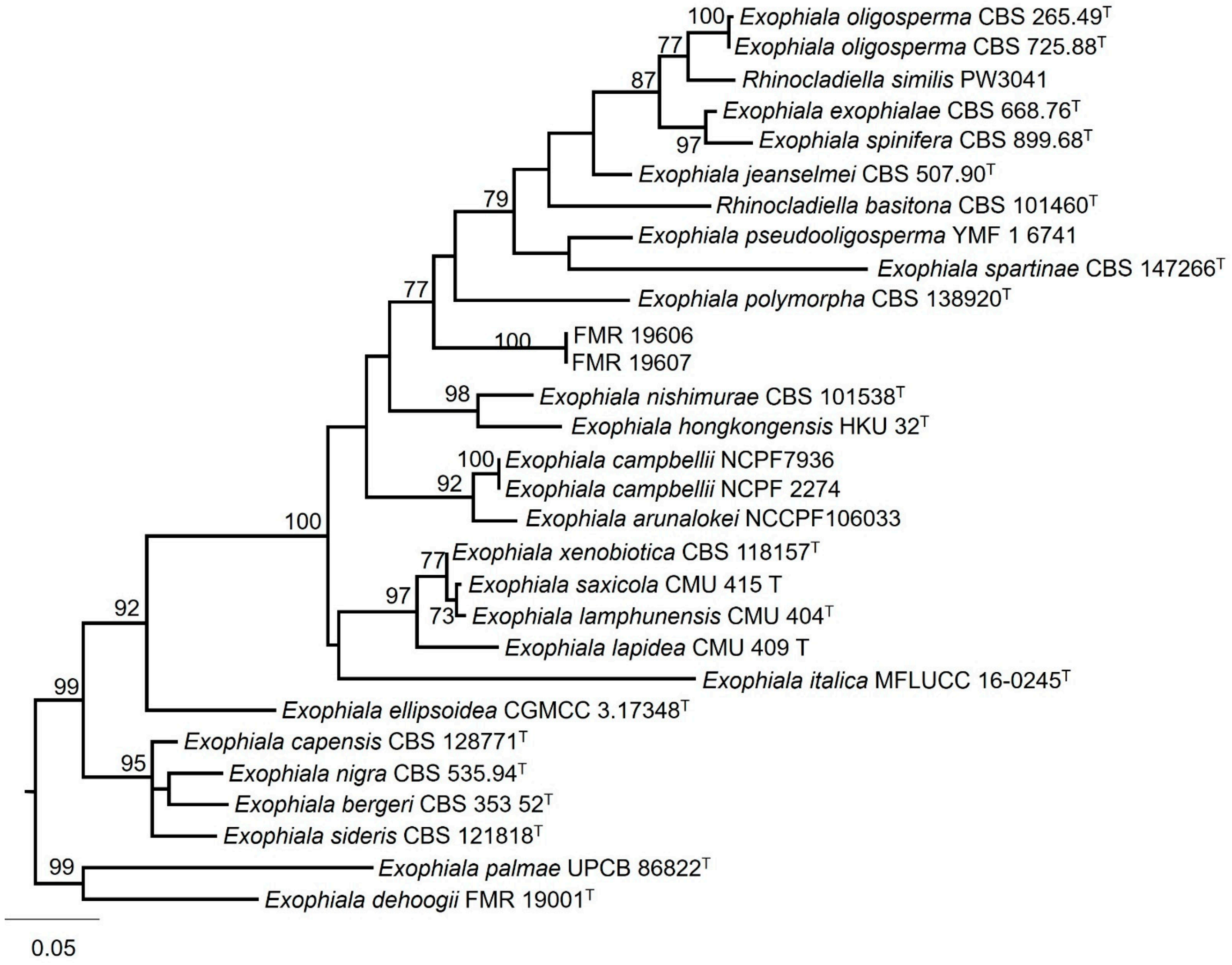

Figure S1: RaxML phylogenetic tree representing the individual ITS alignment of the *Exophiala jeanselmei* clade.

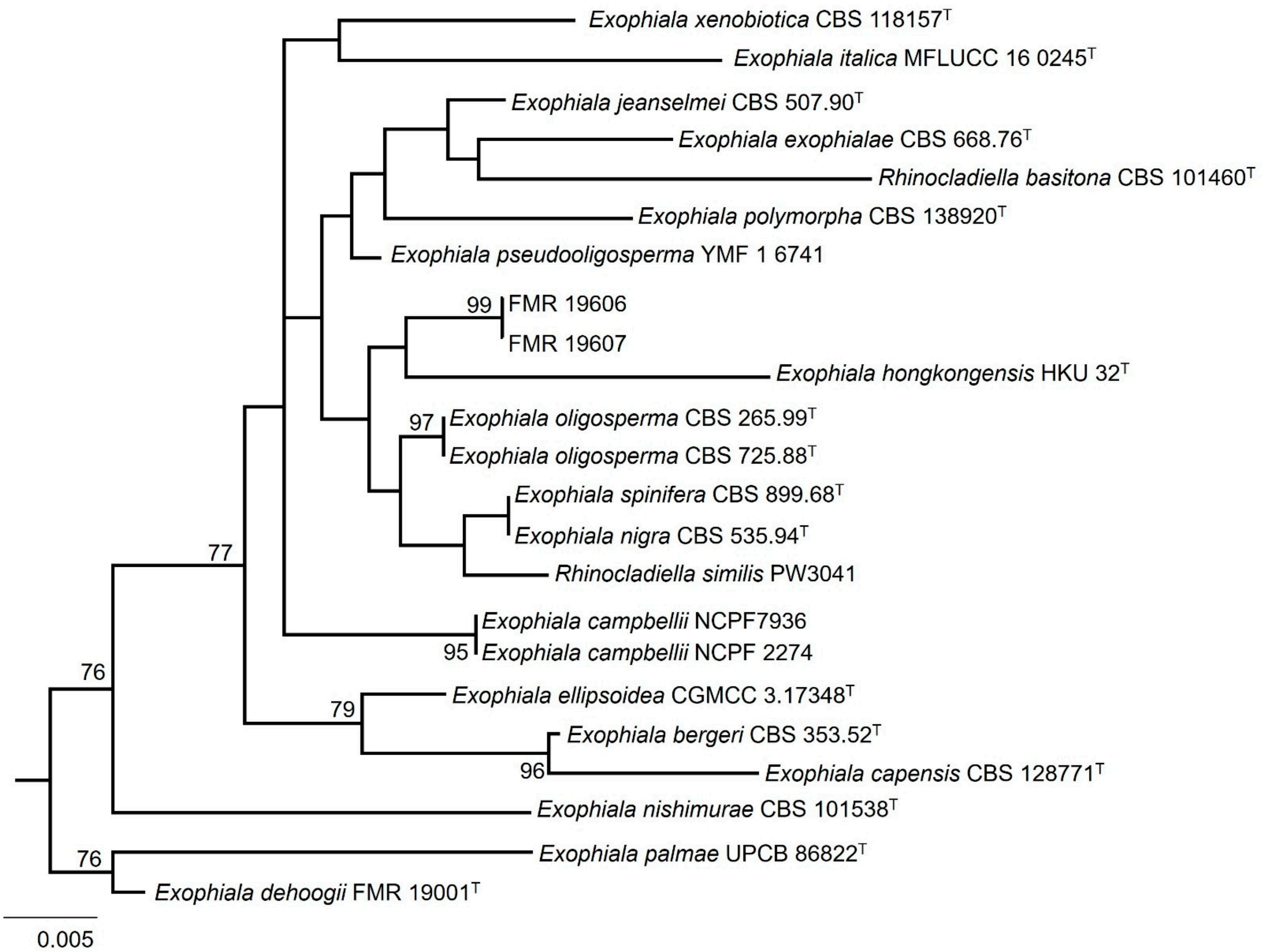

Figure S2: RaxML phylogenetic tree representing the individual LSU alignment of the *Exophiala jeanselmei* clade.

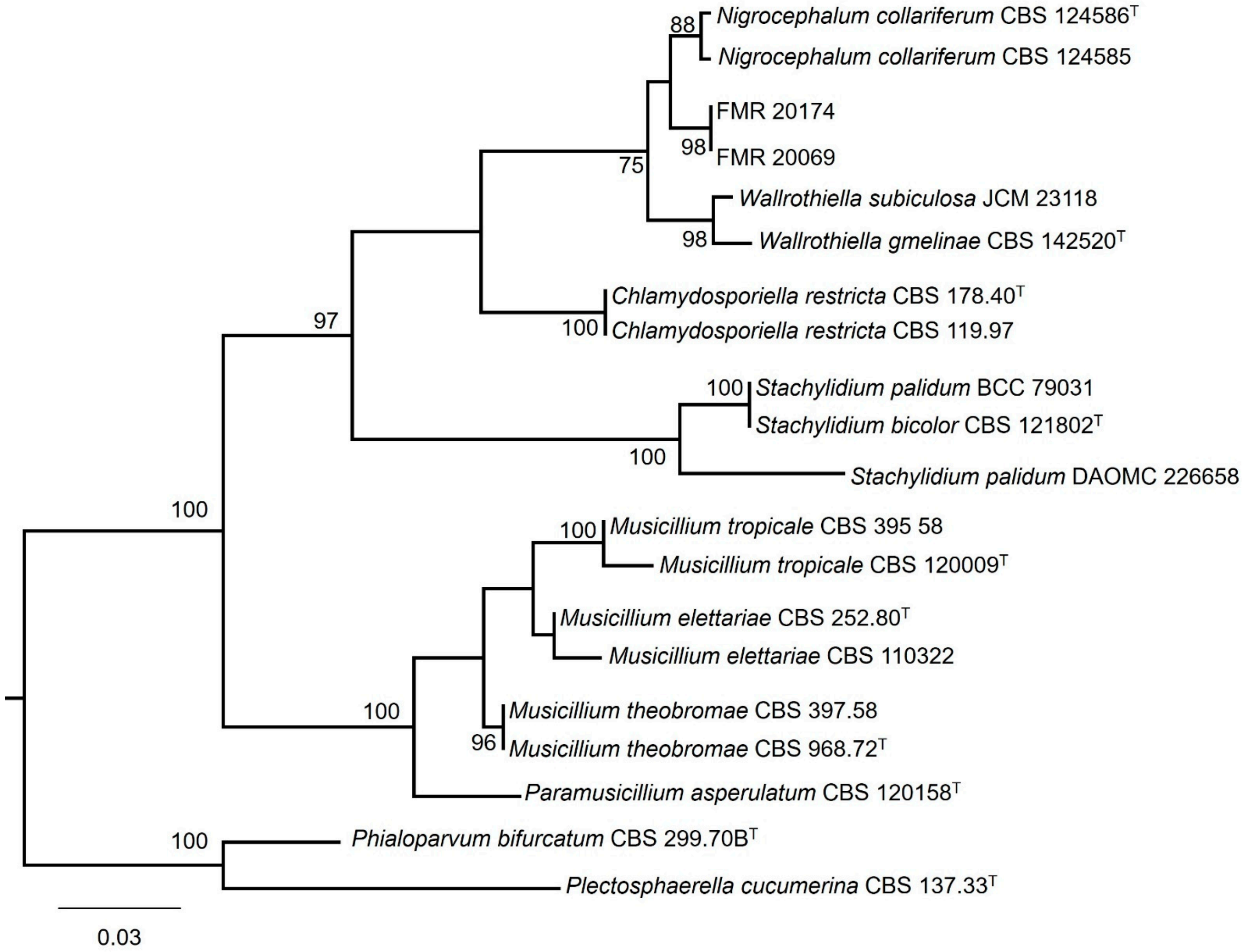

Figure S3: RaxML phylogenetic tree representing the individual ITS alignment of *Nigrocephalum* and representative species of *Plectosphaerellaceae*.

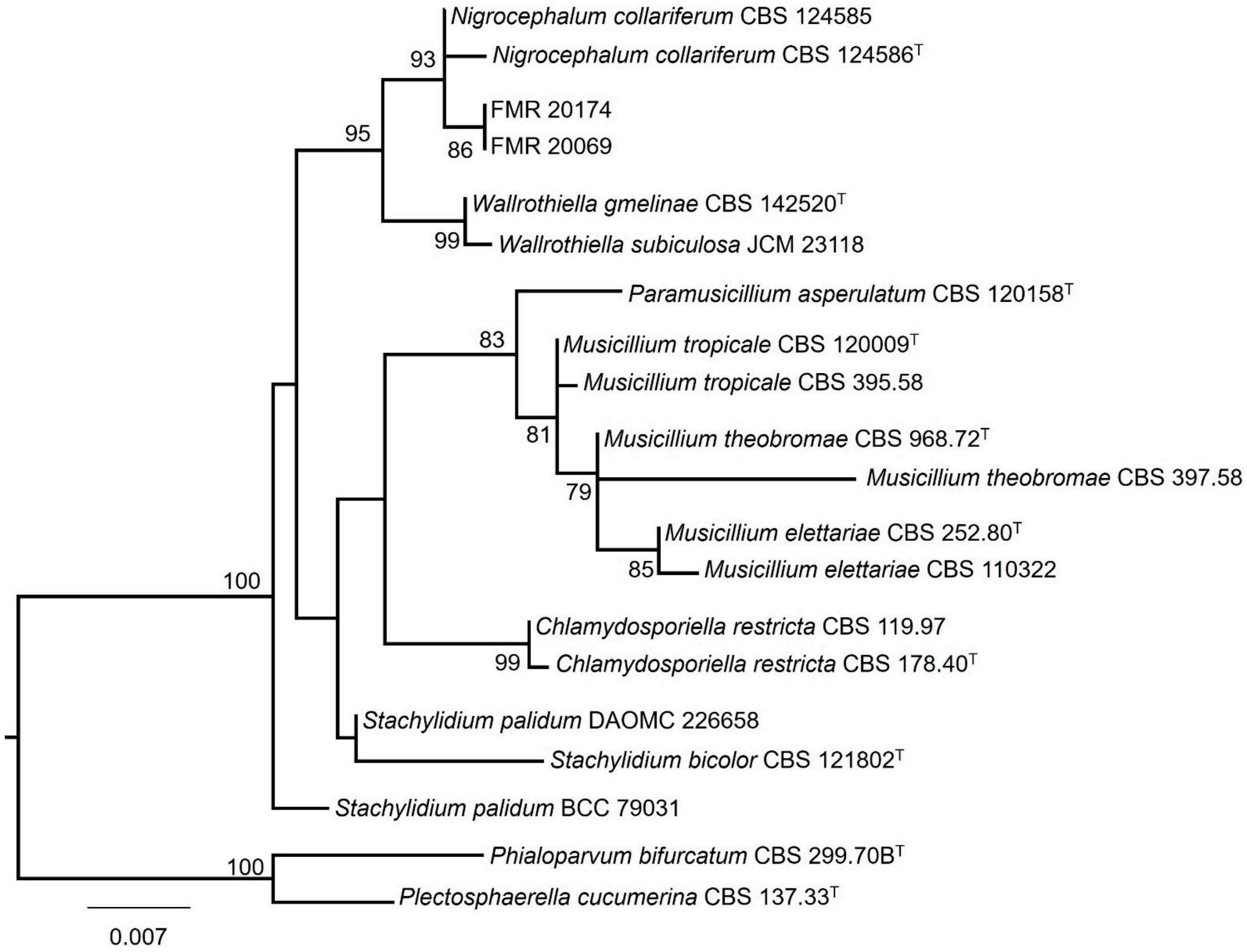

Figure S4: RaxML phylogenetic tree representing the individual LSU alignment of *Nigrocephalum* and representative species of *Plectosphaerellaceae*.

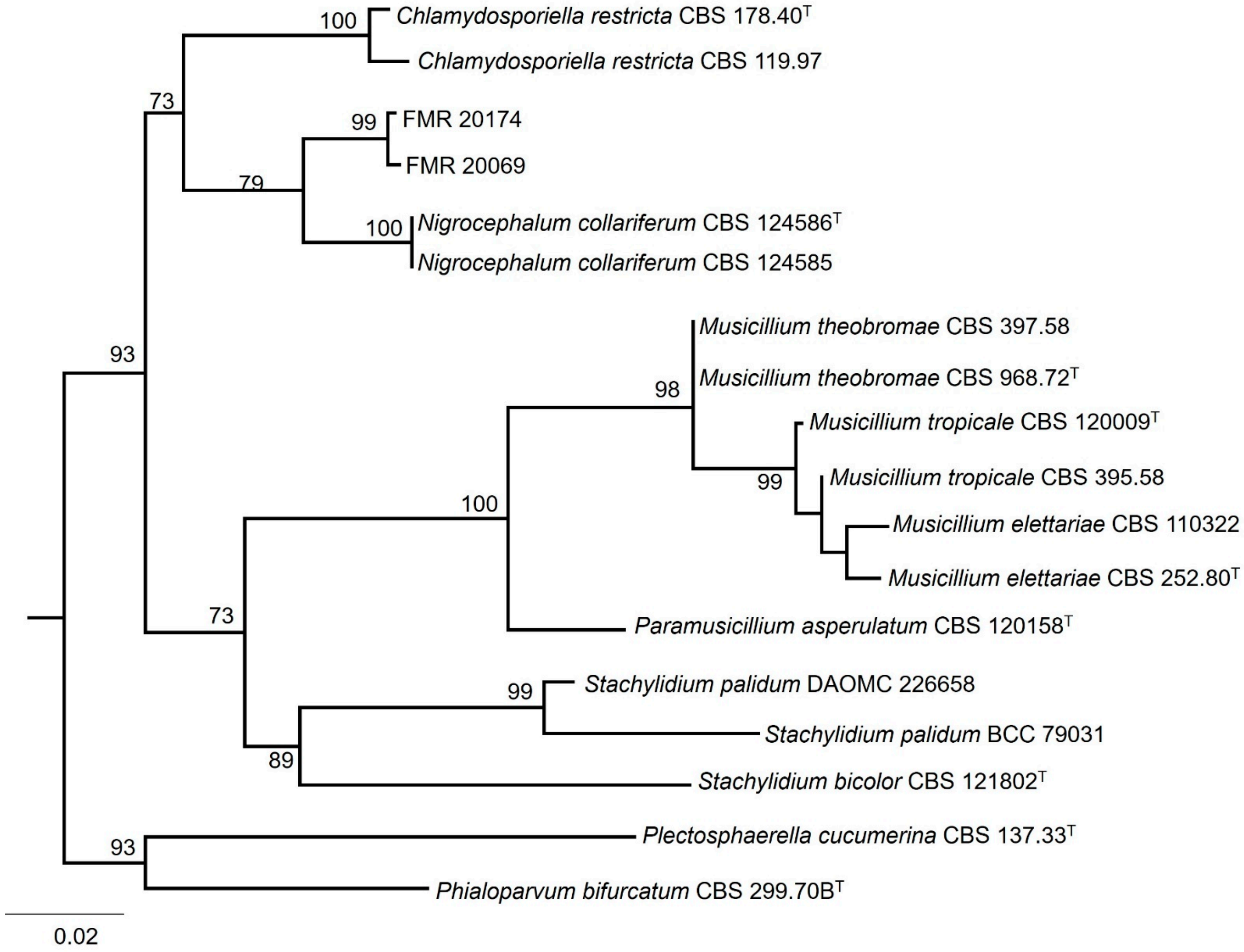

Figure S5: RaxML phylogenetic tree representing the individual *tef*1-α alignment of *Nigrocephalum* and representative species of *Plectosphaerellaceae*.

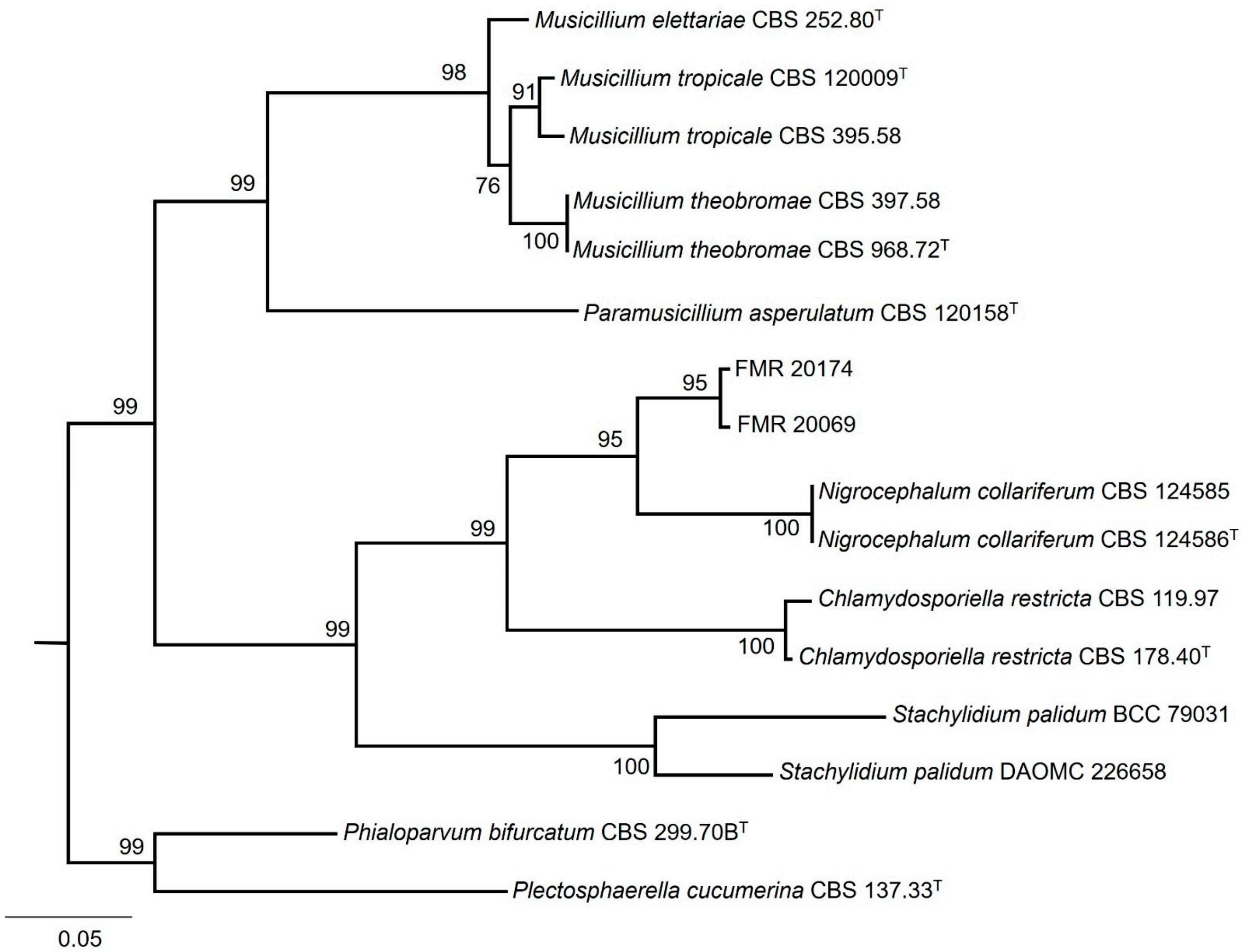

Figure S6: RaxML phylogenetic tree representing the individual *rpb*2 alignment of *Nigrocephalum* and representative species of *Plectosphaerellaceae*.

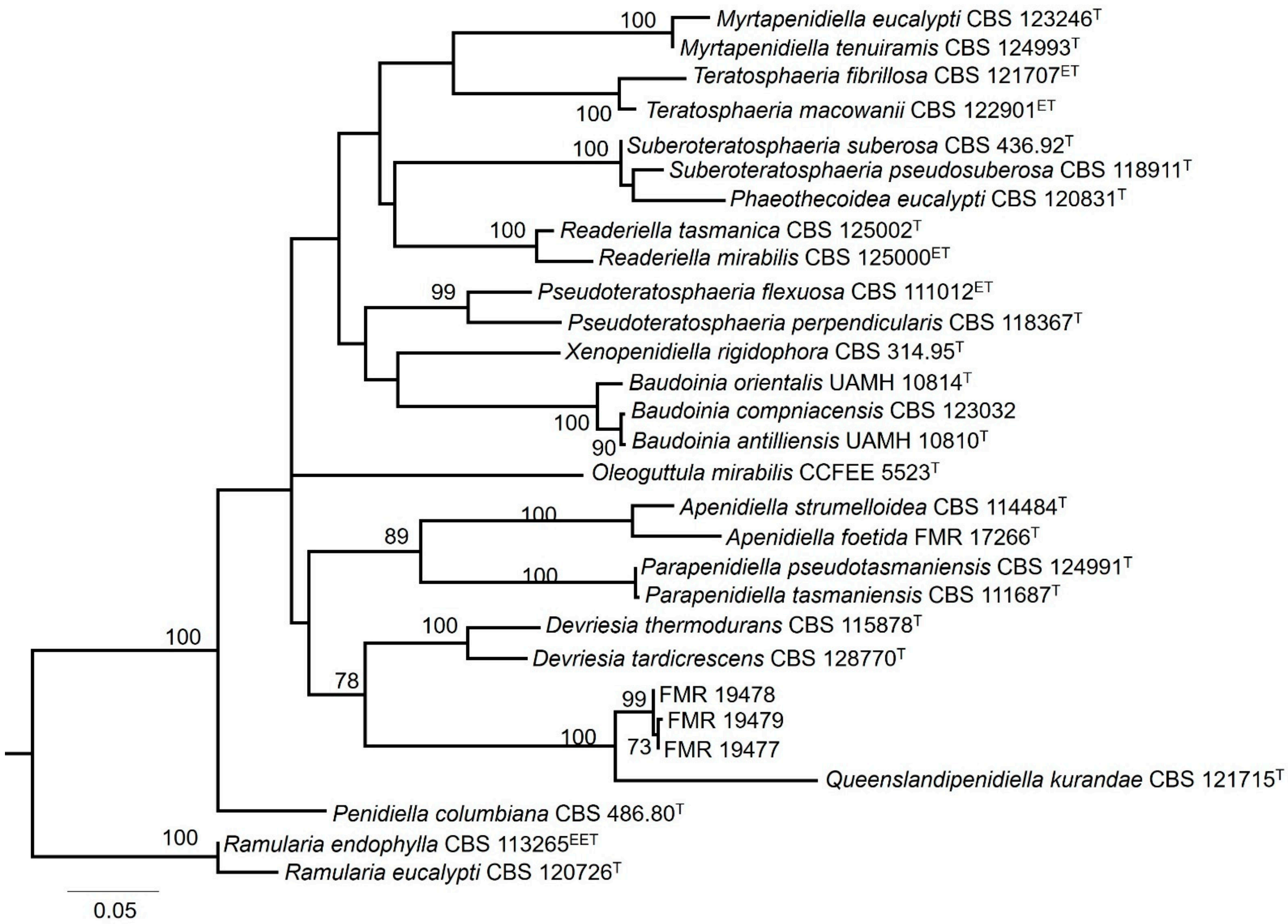

Figure S7: RaxML phylogenetic tree representing the individual ITS alignment of *Queenslandipenidiella* and representative species of *Teratosphaeriaceae*.

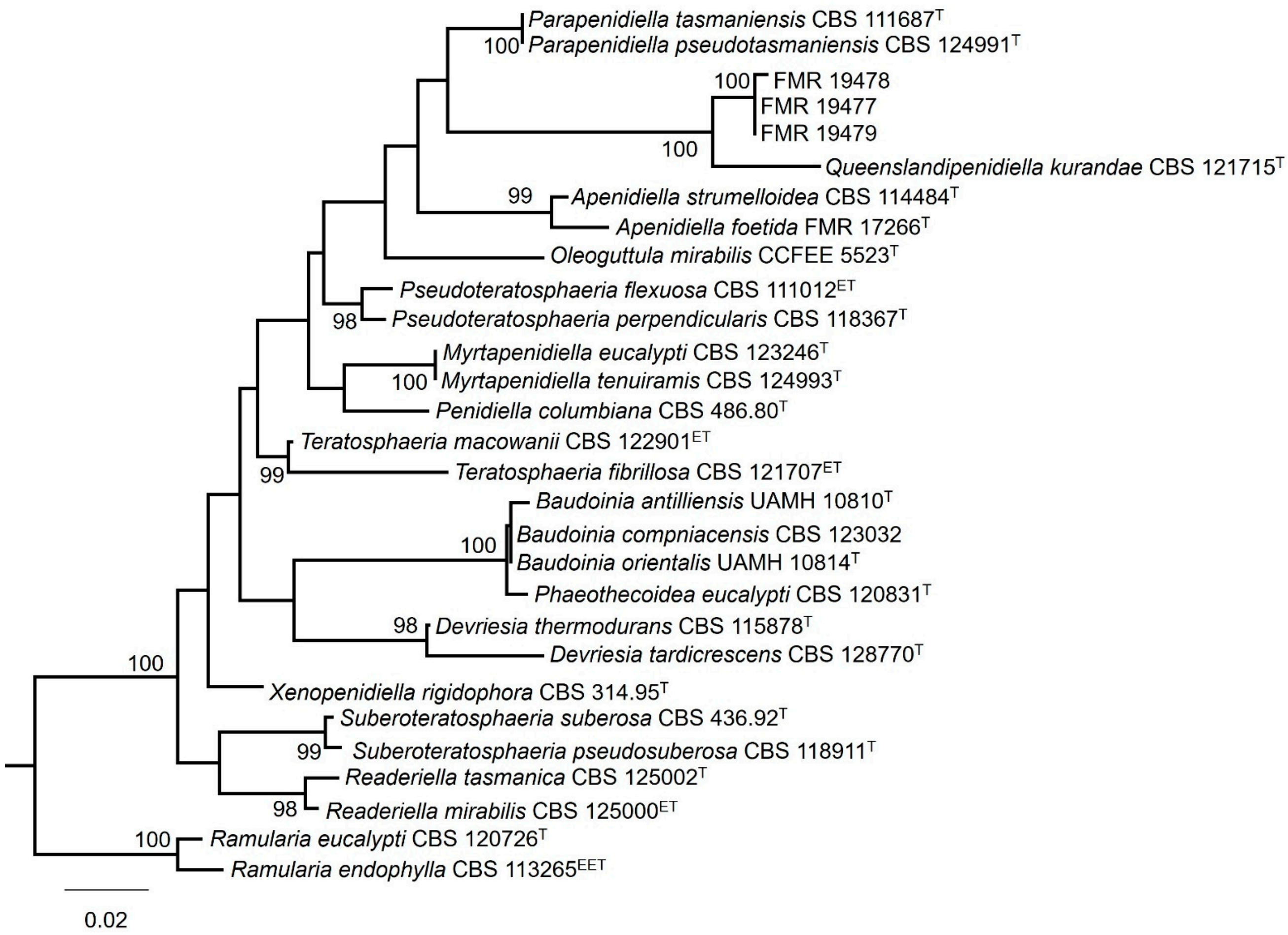

Figure S8: RaxML phylogenetic tree representing the individual LSU alignment of *Queenslandipen-idiella* and representative species of *Teratosphaeriaceae*.

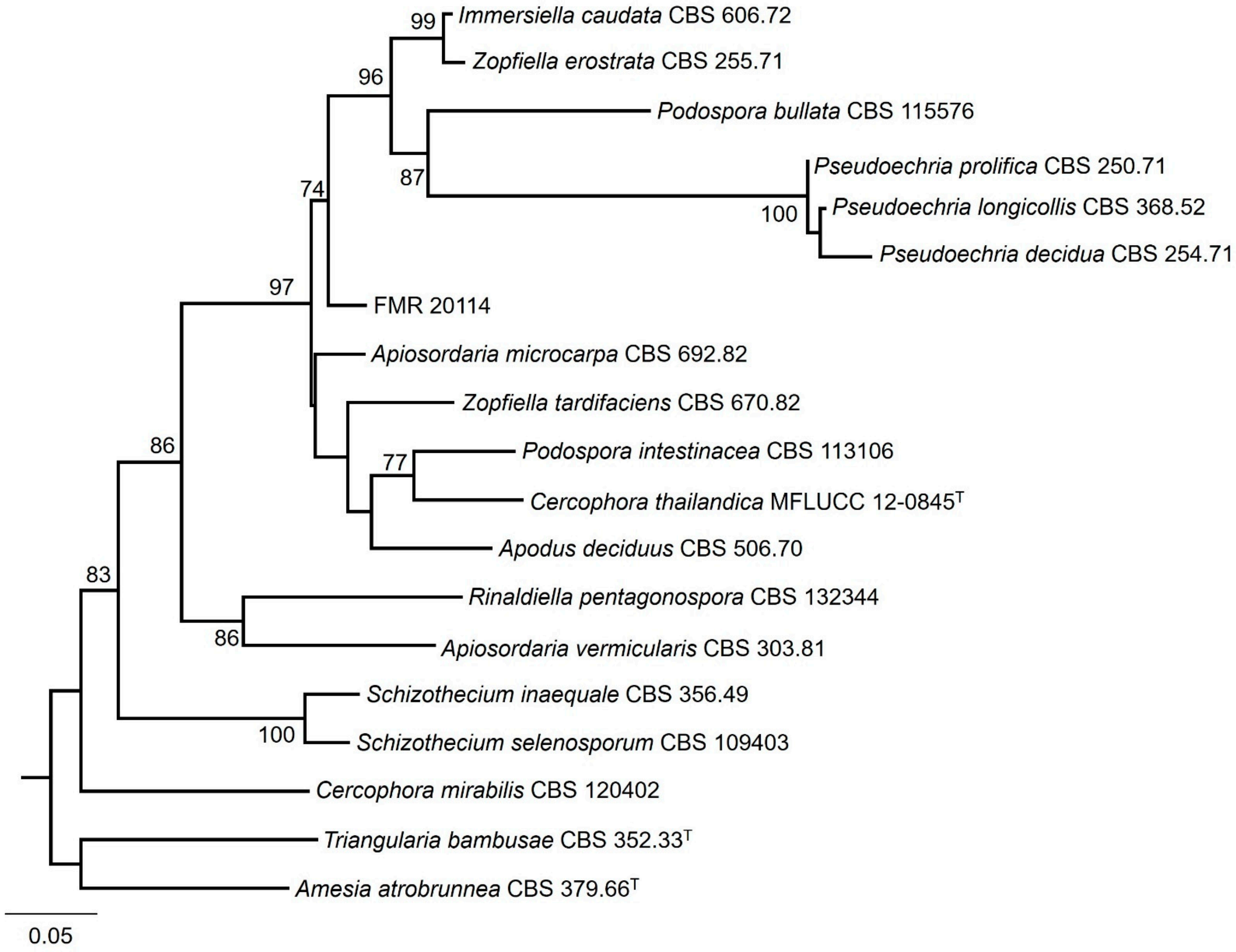

Figure S9: RaxML phylogenetic tree representing the individual ITS alignment of *Schizotheciaceae*.

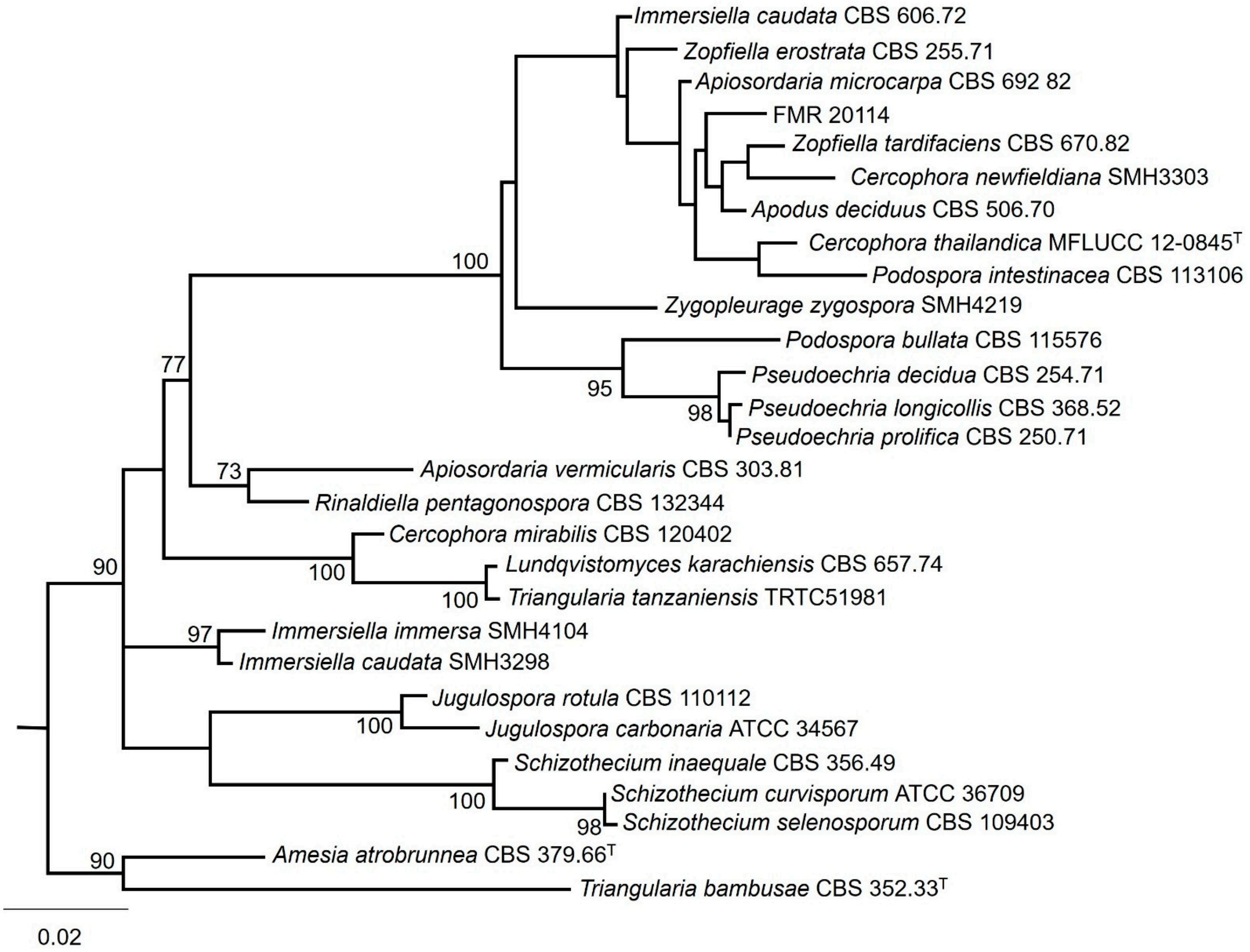

Figure S10: RaxML phylogenetic tree representing the individual LSU alignment of *Schizotheciaceae*.

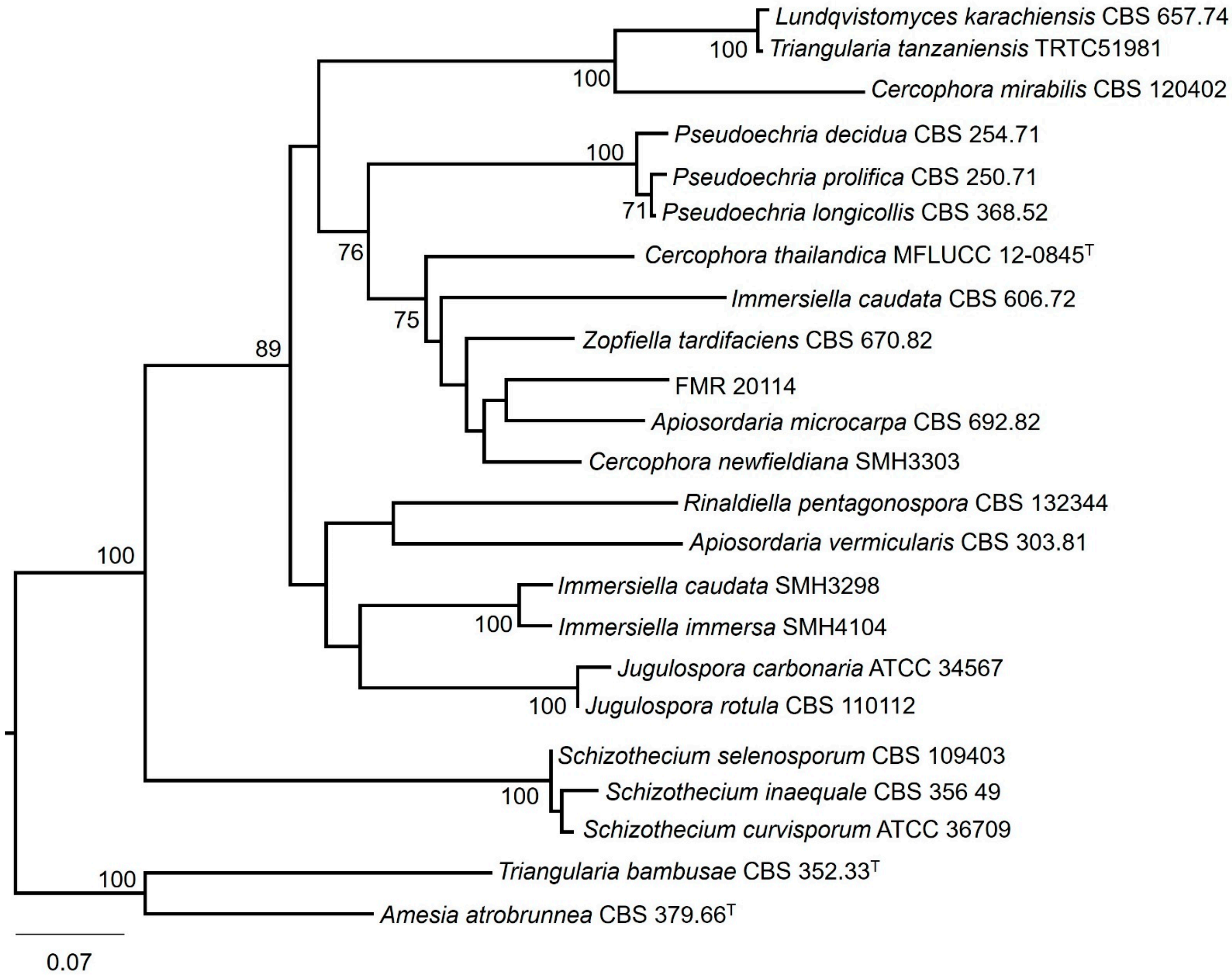

Figure S11: RaxML phylogenetic tree representing the individual *rpb*2 alignment of *Schizotheciaceae*.

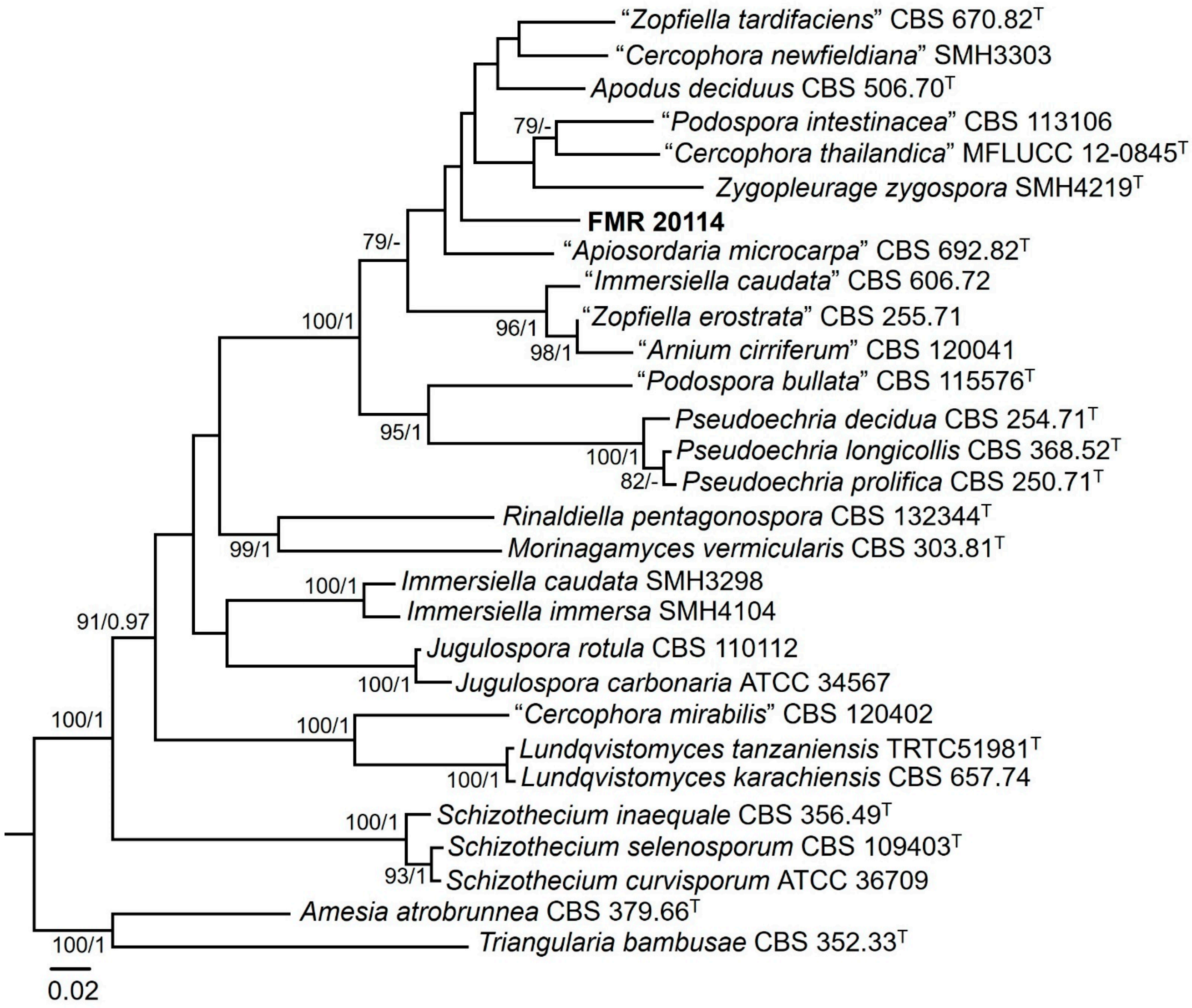

Figure S12: Phylogenetic tree inferred from a concatenated alignment of ITS, LSU and *rpb*2 sequences of 28 strains representing *Schizotheciaceae*. Numbers at the branches indicate support values (RAxML-BS/ BI-PP) above 70%/0.95. The tree is rooted to *Amesia atrobrunnea* CBS 379.66 and *Triangularia bambusae* CBS 352.33. ^{T}indicates ex-type strains. Quote marks indicate strains with unresolved taxonomy. The scale bar represents the expected number of changes per site.
